# Age-specific racial disparities in the incidence of fatal prostate cancer: an analytic deconstruction

**DOI:** 10.1093/jncics/pkaf103

**Published:** 2025-10-23

**Authors:** Mohamed Albirair, Yaw Nyame, Roman Gulati, Ruth Etzioni

**Affiliations:** Department of Global Health, University of Washington, Seattle, WA, United States; Division of Public Health Sciences, Fred Hutchinson Cancer Center, Seattle, WA, United States; Division of Public Health Sciences, Fred Hutchinson Cancer Center, Seattle, WA, United States; Department of Urology, University of Washington Medical Center, Seattle, WA, United States; Division of Public Health Sciences, Fred Hutchinson Cancer Center, Seattle, WA, United States; Division of Public Health Sciences, Fred Hutchinson Cancer Center, Seattle, WA, United States; Department of Health Systems and Population Health, University of Washington, Seattle, WA, United States

## Abstract

**Background:**

Black men in the United States bear a disproportionate share of the prostate cancer (PCa) burden, with more than 50% higher incidence and double the mortality compared with White men. Previous studies have examined racial disparities in the incidence of fatal PCa (fPCa), defined as diagnosis leading to disease-specific death within 10 years, and found that they are greater in younger vs older men. However, the extent to which these trends are driven by disparities in incidence or survival is unknown.

**Methods:**

We conduct a retrospective analysis of data from the Surveillance, Epidemiology, and End Results program over the period 1980-2009 to decompose the incidence of fPCa into incidence and 10-year probability of death and quantify the relative disparities in these metrics by age at diagnosis.

**Findings:**

We find that relative PCa incidence for Black vs White men significantly decreases by 0.116 units with each successive 5-year age group (95% CI = −0.183 to −0.049) but the relative probability of death within 10 years does not differ significantly by age (slope = −0.012, 95% CI = −0.060 to 0.035). Further, this deconstruction is similar before and after the introduction of prostate-specific antigen screening.

**Conclusion:**

We conclude that higher relative incidence of fPCa in young Black men vs young White men appears to be largely driven by their significantly increased incidence of disease. This finding supports investigating targeted screening of Black men beginning at younger ages than White men.

## Introduction

Remarkable racial inequities in health in the United States persist in spite of efforts to reduce them. Progress has been made in understanding underlying factors.^1^^,^^2^ Black men bear a disproportionate share of the cancer burden, having one of the highest death rates of any racial or ethnic group for most cancers.^3-5^ Specifically for prostate cancer (PCa), Black men have more than 50% higher incidence, are more likely to present with distant metastases, and are more than twice as likely to die of the disease compared with non-Hispanic White men.^6^^,^^7^ Although absolute PCa death rates of both Black and White men have declined since the early 1990s, relative rates have remained essentially unchanged.^3^

The reasons for the disparities have not been fully determined. Studies of disease incidence have attributed disparities in PCa incidence to the higher risk of onset among Black men compared to White men^8^ but key drivers of the incidence disparities remain to be determined. A modeling study of PCa natural history found that Black men had a higher risk of preclinical onset at all ages and, given onset, a higher risk of metastatic progression before clinical diagnosis than men in the general population.^9^ Studies of mortality have attributed disparities in mortality to deficiencies in access to care. This explanation is supported by studies conducted in “equal-access health care systems” that show similar mortality outcomes for Black men compared with White men.^6^^,^^10-12^

A challenge with studying racial disparities in the burden of PCa is that screening can lead to overdiagnosis—that is, diagnosis of cancers that would not have been diagnosed without screening. Consequently, disparities in incidence may reflect screening artifact rather than true differences in disease burden. A more meaningful measure may be the incidence of fatal PCa (fPCa), operationalized as incidence of PCa that causes death within 10 years. This measure of PCa burden avoids more complicated comparisons between racial groups due to potentially different rates of overdiagnosis and, because it is defined using both diagnosis and survival,^13^ reflects the burden of the most lethal disease. Kelly et al. examined age-specific fPCa incidence between 1975 and 2002 and concluded that relative disparities were notably higher among younger men.^14^ However, their study did not deconstruct the disparities in fPCa into the portion attributable to incidence vs survival, and did not distinguish between pre- and post-prostate-specific antigen (PSA) eras.

In this study, we further examine the age dependence of racial disparities in fPCa in the United States. Our primary objective is to assess whether age-specific patterns of racial disparities in fPCa incidence are driven by disparities in incidence, fatality (defined as death from PCa within 10 years after diagnosis), or both. Additionally, we partition our analysis into the pre- PSA years (prior to 1990) vs later,^15^ permitting us to evaluate whether mass opportunistic PSA screening impacted racial disparities in fPCa. Our analyses provide new insight into specific screening strategies for achieving greater equity in fPCa between Black and White men in the United States.

## Methods

Our metric for the burden of fPCa was the incidence of disease leading to disease-specific death within a specified period,^13^ here 10 years. We label this measure as the fPCa incidence. The racial disparity in a given group is the ratio of fPCa incidence for Black vs White men in that age group. This measure induces comparability of the cases included across age groups since these cases are constrained to be those who died within 10 years of diagnosis. If, as an alternative, we defined the racial disparity measure as the ratio of mortality for Black vs White men, this would result in a completely different range of survival times for younger and older men, leading to potentially very different disease profiles across age groups.

We included data from the 9 original SEER registries spanning the period between 1975 and 2017, through the National Cancer Institute SEER*Stat software version 8.3.8 (https://www.seer.cancer.gov/seerstat). We obtained point estimates of annual incidence and 10-year net disease-specific survival for 5-year age groups between 45 and 84 years for White and Black men. Net disease-specific survival is defined in SEER as the proportion of patients alive at a certain timepoint subsequent to the diagnosis of their cancer, considering deaths only from the respective cancer and censoring other causes of death (https://www.seer.cancer.gov/statistics/types/survival.html).

To estimate fPCa, we multiplied incidence by one minus the 10-year net cause-specific survival. For each metric, we calculated relative ratios (RRs) for Black vs White men by age. To estimate standard errors for the RRs, we used the delta method (Supplementary Methods 1). We tested for non-constant age-related trends using weighted linear regression models with response variable given by the RR and predictor given by age group.

We defined 2 calendar periods reflecting pre-PSA years and PSA years. For the pre-PSA era, we used the years 1980-1989, reflecting years just prior to the rapid adoption of PSA screening uptake in the United States.^15^ For the PSA era, we used the years 2000-2009, after the period of PSA dissemination. We omit the initial screening dissemination period (1990-1999) to focus on periods of relatively stable diagnostic practices. All calculations are performed separately for both eras. To test for differences in age-related trends in the RR between eras, we included an interaction term for age and era in the weighted linear regression models. The manuscript adheres to the REporting of studies Conducted using Observational Routinely-collected Data guidelines for reporting observational research (Supplementary Methods 2).

## Results

We obtained incidence and survival data on 212 610 and 203 549 cases of PCa in the pre-PSA and PSA eras, respectively.

### Annual PCa incidence rates by age group

**Figure 1.**
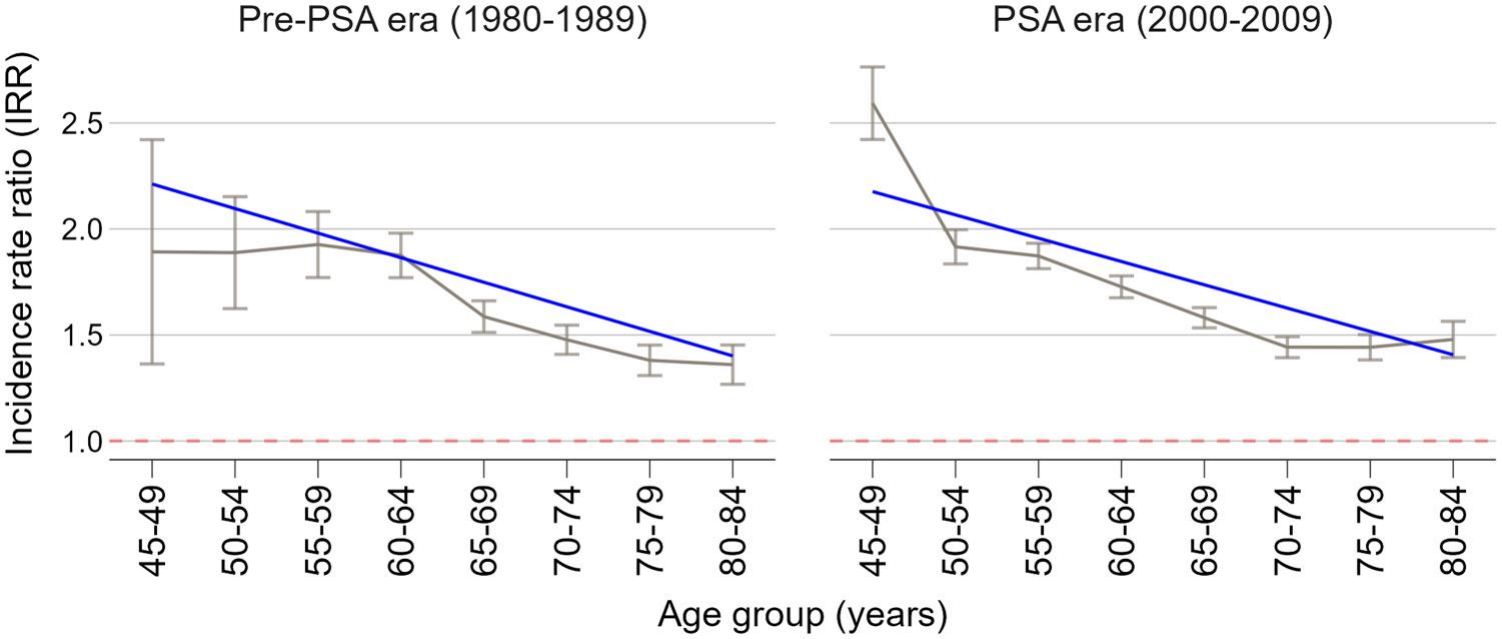
Black-to-White annual prostate cancer incidence rate ratios by era and age group. Figure shows observed rate ratios with 95% CI and fitted trend lines based on weighted linear regression.

Annual PCa Incidence was higher among Black men across all age groups in both calendar periods and generally increased with age but decreased among men 75 years and older in the PSA era, possibly reflecting depletion of the prevalent pool of cases due to screening at younger ages (Figure S1). Figure 1 shows the Black-to-White PCa incidence RRs with 95% CIs for the same age groups in both pre-PSA and PSA eras, with fitted trend lines based on a weighted linear regression model. Table 1 presents the results of the weighted linear regression analysis and shows that incidence RRs decrease with age (slope = −0.116; 95% CI −0.183 to −0.049, *P* = .003) with each consecutive 5-year age group. There is no evidence that incidence RRs by age differed between the pre-PSA and PSA eras (*P* = .9).

**Table 1.** Fitted linear regression of Black-to-White PCa incidence rate ratios by age group at diagnosis and calendar period.

Parameter	Coefficient	95% CI	*P*
Intercept	2.212	1.807 to 2.617	<.001
Age group	−0.116	−0.183 to −0.049	.003
Era	−0.036	−0.484 to 0.413	.865
Age group × era	0.006	−0.071 to 0.083	.87

Reference categories: 45-49 age group and the pre-PSA era.

### 10-year probability of PCa-specific death

The probability of PCa death was consistently higher among Black men in both the pre-PSA and PSA eras and increased with age in both race groups (Figure S2). As expected, the probability of PCa death within 10 years was considerably lower in the PSA era than in the pre-PSA era, reflecting both lead time (the period from screen identification to the anticipated time of clinical diagnosis in the natural disease course) and overdiagnosis (when patients with screen-detected cancers may die from other causes before the time of clinical diagnosis) among detected cases, as well as screening benefit.

**Figure 2.**
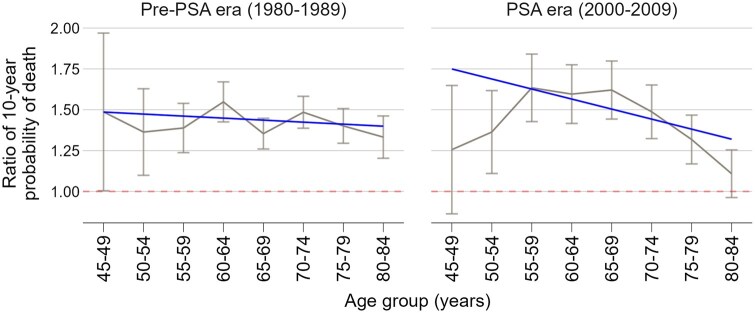
Black-to-White 10-year probability of prostate cancer death ratios by era and age group. Figure shows observed rate ratios with 95% CI and fitted trend lines based on weighted linear regression.

Figure 2 shows the Black-to-White RRs of 10-year probability of PCa death by age group at diagnosis in the pre-PSA and PSA eras. Fitted regression lines suggest increasing ratios with age (Table 2) but the trend is not statistically significant (slope = −0.012; 95% CI = −0.06 to 0.035, *P* = .6). There is no evidence that the trend in the relative probability of PCa death by age differed between the pre-PSA and PSA eras (*P* = .17).

**Table 2.** Fitted linear regression of Black-to-White ratios of 10-year probability of PCa death by age group at diagnosis and calendar period.

Parameter	Coefficient	95% CI	*P*
Intercept	1.486	1.212 to 1.759	<.001
Age group	−0.012	−0.060 to 0.035	.583
Era	0.264	−0.164 to 0.691	.204
Age group × era	−0.049	−0.123 to 0.025	.173

Reference categories: 45-49 age group and the pre-PSA era.

### Incidence of fPCa

**Figure 3.**
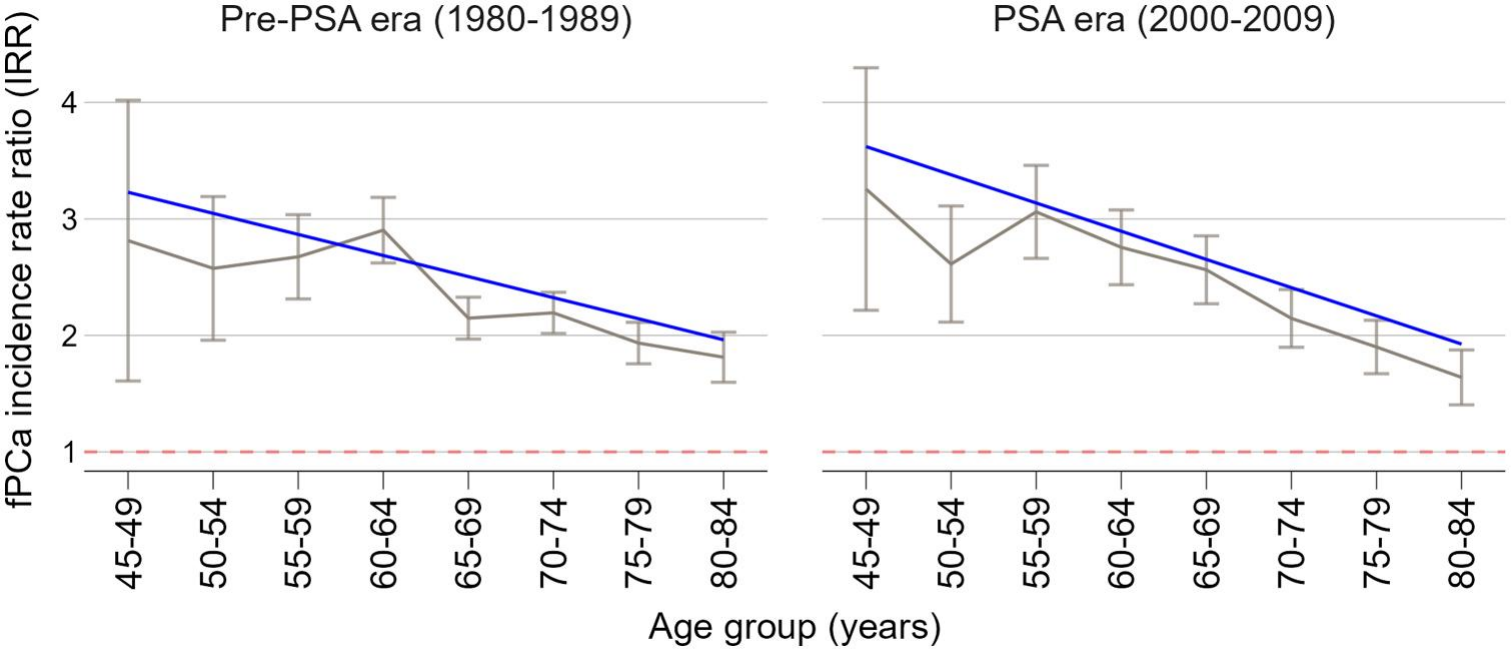
Black-to-White annual incidence rate ratios of fPCa incidence by era and age group. Figure shows observed rate ratios with 95% CI and fitted trend lines based on weighted linear regression.

fPCa incidence estimates were higher among Black men in both eras, were higher at older ages in both race groups, and were lower in both race groups in the PSA era (Figure S3). Figure 3 shows fPCa incidence rate ratios by age group at diagnosis, calendar period, and race group. Table 3 provides the results of the linear regression fit to the fPCa incidence rate ratios and shows that the relative incidence declines significantly (slope = −0.181; 95% CI −0.26 to −0.102; *P* < .001) with each consecutive 5-year age group. There is no evidence that Black-to-White fPCa incidence RRs changed with age differentially between the pre-PSA and PSA eras (*P* = .3).

**Table 3.** Fitted linear regression of Black-to-White incidence rate ratios of fPCa by age group at diagnosis and calendar period.

Parameter	Coefficient	95% CI	*P*
Intercept	3.229	2.752 to 3.706	<.001
Age group	−0.181	−0.260 to −0.102	<.001
Era	0.392	−0.308 to 1.093	.246
Age group × era	−0.061	−0.176 to 0.054	.269

Reference categories: 45-49 age group and the pre-PSA era.

## Discussion

This study examines racial disparities in fPCa incidence and its association with age at diagnosis. It builds on the study by Kelly et al.^14^ by examining the individual incidence and survival components and evaluating possible differences between the pre-PSA and PSA eras. Given the dramatic incidence differential between younger and older men, we felt that relative disparities would be more consistently interpretable across the age continuum than absolute disparities. We found strong evidence that higher fPCa incidence in Black men vs White men at young ages was largely determined by the higher PCa incidence—and not the higher probability of PCa death—in Black men at these ages. We also found that the age dependence in fPCa disparities did not change notably between the pre-PSA and PSA eras.

Screening using the PSA test has been shown to be effective in reducing PCa mortality.^16-18^ A modeling study by Gulati et al. showed that earlier and more frequent PSA-based screening among high-risk men could potentially reduce the mortality disparity through earlier diagnosis of tumors that otherwise may become metastatic.^19^ Shenoy et al. studied differences in PCa epidemiology and outcomes between Caucasian and African American men and concluded that separate PCa screening guidelines are likely necessary to help save the lives of African Americans.^20^ Tsodikov et al. recommended that screening should be initiated at an earlier age for Black men compared to the average-risk population.^9^ Etzioni and Nyame concluded that Black men should be considered eligible for screening 10 years before the rest of the general population.^21^ In addition, Nyame et al. showed that screening earlier and more frequently among Black men is expected to achieve greater projected mortality reductions than standard practices and could narrow the disparity gap.^22^ Garraway et al. estimated 30% relative mortality reduction with baseline testing at ages 40 and 45 for Black men who elect to regular PSA screening without substantially increasing overdiagnosis.^23^

Results of this analysis suggest that there is merit in proposals to lower the age to start screening for Black men. Starting screening earlier for Black men could bring the survival benefits of screening to younger patients, reducing the excessive disparity in fPCa among younger men. Other interventions to reduce fatality among younger Black cases, such as more frequent screening and improved uptake and quality of curative treatment, could also contribute. Black men have historically selected curative treatments, such as surgery for localized disease, significantly less frequently than White men^24^^,^^25^ and also may be subject to poorer quality treatment.^26^^,^^27^ Across all 3 metrics in our study, there was no significant difference in the age-specific disparities between the pre- and PSA eras, suggesting that no additional benefit was offered to either race group.

Patterns of age-related disparities in breast cancer mortality have also been informative about disease biology and potential directions for intervention. In their analysis of age-related disparities in breast cancer mortality, San Miguel et al. reported more pronounced Black-White survival disparities in younger vs older patients, and suggested that this may reflect more aggressive disease diagnosed among younger non-Hispanic Black women.^28^ Hendrick et al. examined risks of advanced-stage breast cancer among women younger than 50 years and found that minority women collectively had a 58% higher risk compared with non-Hispanic White women. They concluded that this evident disparity justifies the need for more aggressive treatment among minority women with breast cancer.^29^ Similar to intensified breast cancer screening in Black women, targeted PSA screening in Black men should involve close monitoring of outcomes to assess added benefit.

The use of SEER registry data is a significant strength as it covers a large catchment area including diverse geographic regions and demographic groups across the United States. Our study has several limitations. First, we used an arbitrary interval to define fPCa, namely 10 years, for both pre-PSA and PSA eras. This is a relatively short time frame for examining PCa survival, which can extend considerably beyond 10 years for a substantial fraction of cases, particularly in the PSA era. In a sensitivity analysis, we extended the duration defining fPCa to 17 years (based on SEER data available for our defined PSA era) and reached a similar conclusion, that the main driver of fPCa disparity trends by age are the age-related disparities in disease incidence rather than fatality (Figures S4 and S5 and Table S1).

Second, the generalizability of our findings is limited by the fact that SEER registries represent US demographic groups, with oversampling for African Americans along with other racial groups. Kuo and Mobley explored the generalizability of SEER registries to the cancer population in the United States and reported that it had greater economic disadvantage and greater minority diversity among the population, and that should be taken into consideration when generalizing findings to the entire United States.^30^ Finally, we focused on disparities in the relative scale instead of the absolute scale. We note that, although we observed a dramatic relative disparity in fPCa among young men, these disparities amount to small absolute differences in fPCa rates. Indeed, as one might expect, the absolute disparity in fPCa incidence is far greater for older men than for younger men.

## Conclusion

In conclusion, the pattern of age-specific disparities in fPCa incidence appears to be driven more by disparities in incidence than by disparities in fatality, and these patterns of fPCa incidence did not substantially change between the pre-PSA and PSA eras even though both incidence and disease-specific survival are significantly increased in the PSA era. These findings are broadly consistent with advocacy based on other studies to offer screening to Black men at younger ages compared with White counterparts and to provide them with access to appropriate curative treatment once diagnosed.

## Supplementary Material

pkaf103_Supplementary_Data

## Data Availability

The data analyzed in this study were publicly obtained from the Surveillance, Epidemiology, and End Results (SEER) program. The R code necessary to reproduce the analyses, along with instructions for data download, is available in the GitHub repository: https://github.com/Mohamed-Albirair/age_specific_fpca_racial_disparities/tree/main.
