# Supplementary material for: Age-specific racial disparities in the incidence of fatal prostate cancer: an analytic deconstruction
Source: JNCI Cancer Spectr. 2025 Oct 23;9(6):pkaf103. doi: 10.1093/jncics/pkaf103 (PMC12615997; doi:10.1093/jncics/pkaf103)
Supplement: pkaf103_Supplementary_Data [file pkaf103_supplementary_data.zip › Supplementary Material.docx]

# **Supplementary Material**

## Supplementary Methods 1: Estimating the Standard Error of an Estimate Using the Delta Method

The Delta Method is a widely used statistical technique for approximating the standard error of a function of one or more random variables. It provides a useful approximation for their standard errors, especially when the estimates are asymptotically normal^[[1]](#footnote-1)^.

In the case where an estimate is a product of two estimates with known variances ($G\left( y_{1},y_{2} \right)=y_{1}\times y_{2}$), then the variance of the product is obtained by the formula:

$$var\left( G\left( y_{1},y_{2} \right) \right)=\left( \mu_{1} \right)^{2}\times var\left( y_{2} \right)+\left( \mu_{2} \right)^{2}\times var\left( y_{1} \right)$$

Where $G$ is the product of estimates $y_{1}$ and $y_{2}$; and $mu_{1}$ and $mu_{2}$ are means of $y_{1}$ and $y_{2}$, respectively.

On the other hand, in the case where the estimate is the quotient of two estimates with known variances ($G\left( y_{1},y_{2} \right)=y_{1}\div y_{2}$), then the variance of the product is obtained by the formula:

$$var\left( G\left( y_{1},y_{2} \right) \right)=\frac{1}{\left( \mu_{2} \right)^{2}}\times var\left( y_{1} \right)+\frac{\left( \mu_{1} \right)^{2}}{\left( \mu_{2} \right)^{4}}\times var\left( y_{2} \right)$$

Where $G$ is the quotient of estimates $y_{1}$ and $y_{2}$; and $mu_{1}$ and $mu_{2}$ are means of $y_{1}$ and $y_{2}$, respectively.

## Supplementary Methods 2: Completed STROBE/RECORD Reporting Checklist

**The RECORD statement – checklist of items, extended from the STROBE statement, that should be reported in observational studies using routinely collected health data.**

|  | **Item No.** | **STROBE items** | **Location in manuscript where items are reported** | **RECORD items** | **Location in manuscript where items are reported** |
| --- | --- | --- | --- | --- | --- |
| **Title and abstract** | | | | | |
|  | 1 | (a) Indicate the study’s design with a commonly used term in the title or the abstract (b) Provide in the abstract an informative and balanced summary of what was done and what was found |  | RECORD 1.1: The type of data used should be specified in the title or abstract. When possible, the name of the databases used should be included.  RECORD 1.2: If applicable, the geographic region and timeframe within which the study took place should be reported in the title or abstract.  RECORD 1.3: If linkage between databases was conducted for the study, this should be clearly stated in the title or abstract. | Line 25, we explicitly state the name and type of data. |
| **Introduction** | | | | | |
| Background rationale | 2 | Explain the scientific background and rationale for the investigation being reported |  |  | Lines 39-68. |
| Objectives | 3 | State specific objectives, including any prespecified hypotheses |  |  | Line 70, we state the objective, but since this is an exploratory analysis, we did not state any hypotheses. |
| **Methods** | | | | | |
| Study Design | 4 | Present key elements of study design early in the paper |  |  | Line 87 |
| Setting | 5 | Describe the setting, locations, and relevant dates, including periods of recruitment, exposure, follow-up, and data collection |  |  | Line 87. Recruitment, exposure, and follow-up periods are not applicable. |
| Participants | 6 | *(a) Cohort study* - Give the eligibility criteria, and the sources and methods of selection of participants. Describe methods of follow-up  *Case-control study* - Give the eligibility criteria, and the sources and methods of case ascertainment and control selection. Give the rationale for the choice of cases and controls  *Cross-sectional study* - Give the eligibility criteria, and the sources and methods of selection of participants  *(b) Cohort study* - For matched studies, give matching criteria and number of exposed and unexposed  *Case-control study* - For matched studies, give matching criteria and the number of controls per case |  | RECORD 6.1: The methods of study population selection (such as codes or algorithms used to identify subjects) should be listed in detail. If this is not possible, an explanation should be provided.  RECORD 6.2: Any validation studies of the codes or algorithms used to select the population should be referenced. If validation was conducted for this study and not published elsewhere, detailed methods and results should be provided.  RECORD 6.3: If the study involved linkage of databases, consider use of a flow diagram or other graphical display to demonstrate the data linkage process, including the number of individuals with linked data at each stage. | Line 88. We included all populations in the registry data, we imposed no selection criteria other than male sex and age being between 45 and 80. |
| Variables | 7 | Clearly define all outcomes, exposures, predictors, potential confounders, and effect modifiers. Give diagnostic criteria, if applicable. |  | RECORD 7.1: A complete list of codes and algorithms used to classify exposures, outcomes, confounders, and effect modifiers should be provided. If these cannot be reported, an explanation should be provided. | Not applicable |
| Data sources/ measurement | 8 | For each variable of interest, give sources of data and details of methods of assessment (measurement).  Describe comparability of assessment methods if there is more than one group |  |  | Lines 87-108 |
| Bias | 9 | Describe any efforts to address potential sources of bias |  |  | Not applicable since we analyze all data within the defined period |
| Study size | 10 | Explain how the study size was arrived at |  |  | Line 87 |
| Quantitative variables | 11 | Explain how quantitative variables were handled in the analyses. If applicable, describe which groupings were chosen, and why |  |  | Lines 87-108 |
| Statistical methods | 12 | (a) Describe all statistical methods, including those used to control for confounding  (b) Describe any methods used to examine subgroups and interactions  (c) Explain how missing data were addressed  (d) *Cohort study* - If applicable, explain how loss to follow-up was addressed  *Case-control study* - If applicable, explain how matching of cases and controls was addressed  *Cross-sectional study* - If applicable, describe analytical methods taking account of sampling strategy  (e) Describe any sensitivity analyses |  |  | Lines 87-108 |
| Data access and cleaning methods |  | .. |  | RECORD 12.1: Authors should describe the extent to which the investigators had access to the database population used to create the study population.  RECORD 12.2: Authors should provide information on the data cleaning methods used in the study. |  |
| Linkage |  | .. |  | RECORD 12.3: State whether the study included person-level, institutional-level, or other data linkage across two or more databases. The methods of linkage and methods of linkage quality evaluation should be provided. | Not applicable |
| **Results** | | | | | |
| Participants | 13 | (a) Report the numbers of individuals at each stage of the study (*e.g.*, numbers potentially eligible, examined for eligibility, confirmed eligible, included in the study, completing follow-up, and analysed)  (b) Give reasons for non-participation at each stage.  (c) Consider use of a flow diagram |  | RECORD 13.1: Describe in detail the selection of the persons included in the study (*i.e.,* study population selection) including filtering based on data quality, data availability and linkage. The selection of included persons can be described in the text and/or by means of the study flow diagram. | Not applicable |
| Descriptive data | 14 | (a) Give characteristics of study participants (*e.g.*, demographic, clinical, social) and information on exposures and potential confounders  (b) Indicate the number of participants with missing data for each variable of interest  (c) *Cohort study* - summarise follow-up time (*e.g.*, average and total amount) |  |  | Line 109. Missing data, if any, are minimal, and are handled by SEER. |
| Outcome data | 15 | *Cohort study* - Report numbers of outcome events or summary measures over time  *Case-control study* - Report numbers in each exposure category, or summary measures of exposure  *Cross-sectional study* - Report numbers of outcome events or summary measures |  |  | Lines 108-142 |
| Main results | 16 | (a) Give unadjusted estimates and, if applicable, confounder-adjusted estimates and their precision (e.g., 95% confidence interval). Make clear which confounders were adjusted for and why they were included  (b) Report category boundaries when continuous variables were categorized  (c) If relevant, consider translating estimates of relative risk into absolute risk for a meaningful time period |  |  | Lines 108-142. We do not translate relative metrics to absolute metrics, but acknowledge implications at the end of the discussion section. |
| Other analyses | 17 | Report other analyses done—e.g., analyses of subgroups and interactions, and sensitivity analyses |  |  | Line 194 |
| **Discussion** | | | | | |
| Key results | 18 | Summarise key results with reference to study objectives |  |  | 109-147 |
| Limitations | 19 | Discuss limitations of the study, taking into account sources of potential bias or imprecision. Discuss both direction and magnitude of any potential bias |  | RECORD 19.1: Discuss the implications of using data that were not created or collected to answer the specific research question(s). Include discussion of misclassification bias, unmeasured confounding, missing data, and changing eligibility over time, as they pertain to the study being reported. | 189-208 |
| Interpretation | 20 | Give a cautious overall interpretation of results considering objectives, limitations, multiplicity of analyses, results from similar studies, and other relevant evidence |  |  | 143-208 |
| Generalisability | 21 | Discuss the generalisability (external validity) of the study results |  |  | 204-208 |
| **Other Information** | | | | | |
| Funding | 22 | Give the source of funding and the role of the funders for the present study and, if applicable, for the original study on which the present article is based |  |  | Funding section |
| Accessibility of protocol, raw data, and programming code |  | .. |  | RECORD 22.1: Authors should provide information on how to access any supplemental information such as the study protocol, raw data, or programming code. | Will be provided |

*Reference: Benchimol EI, Smeeth L, Guttmann A, Harron K, Moher D, Petersen I, Sørensen HT, von Elm E, Langan SM, the RECORD Working Committee. The REporting of studies Conducted using Observational Routinely-collected health Data (RECORD) Statement. *PLoS Medicine* 2015; in press.

*Checklist is protected under Creative Commons Attribution ([CC BY](http://creativecommons.org/licenses/by/4.0/)) license.

## Figure S1


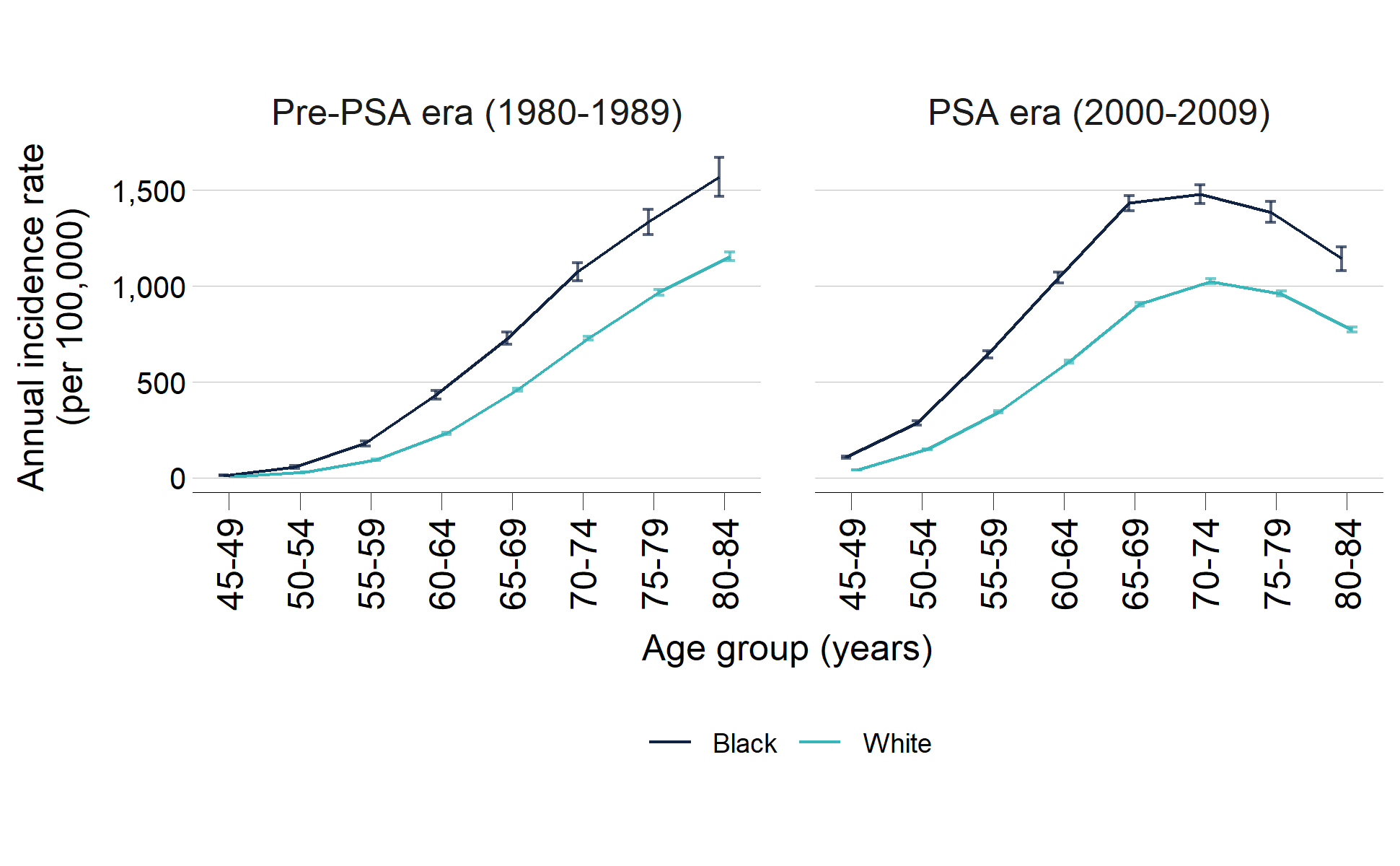


Annual prostate cancer incidence rates by era, race, and age group. Figure shows observed incidence rates per 100,000 individuals with 95% confidence intervals.

## Figure S2


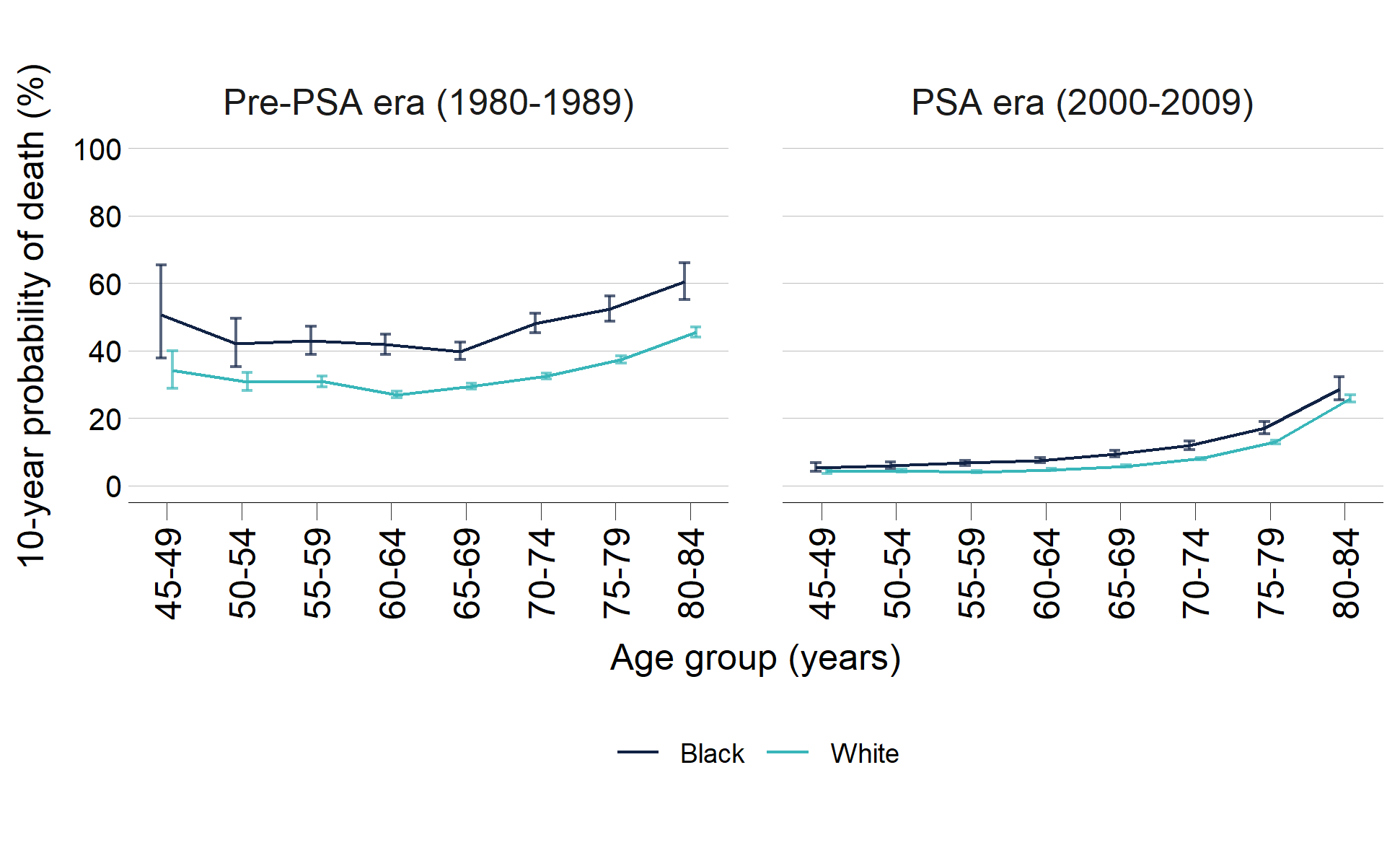


10-year prostate cancer probability of death by era, race, and age group. Figure shows observed incidence rates with 95% confidence intervals.

## Figure S3


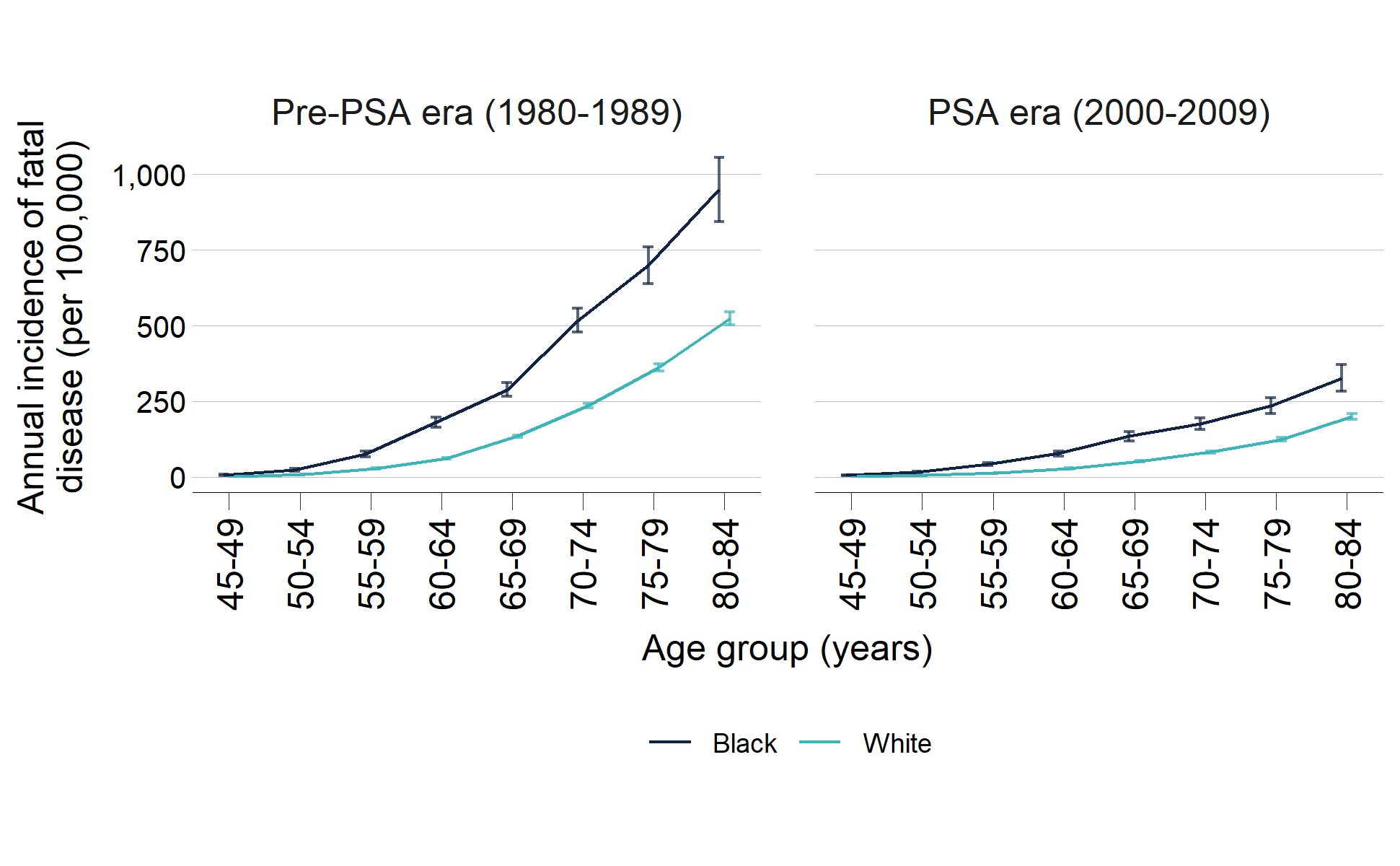


Annual incidence rates of fatal prostate cancer by era, race, and age group. Figure shows observed incidence rates with 95% confidence intervals.

## Figure S4


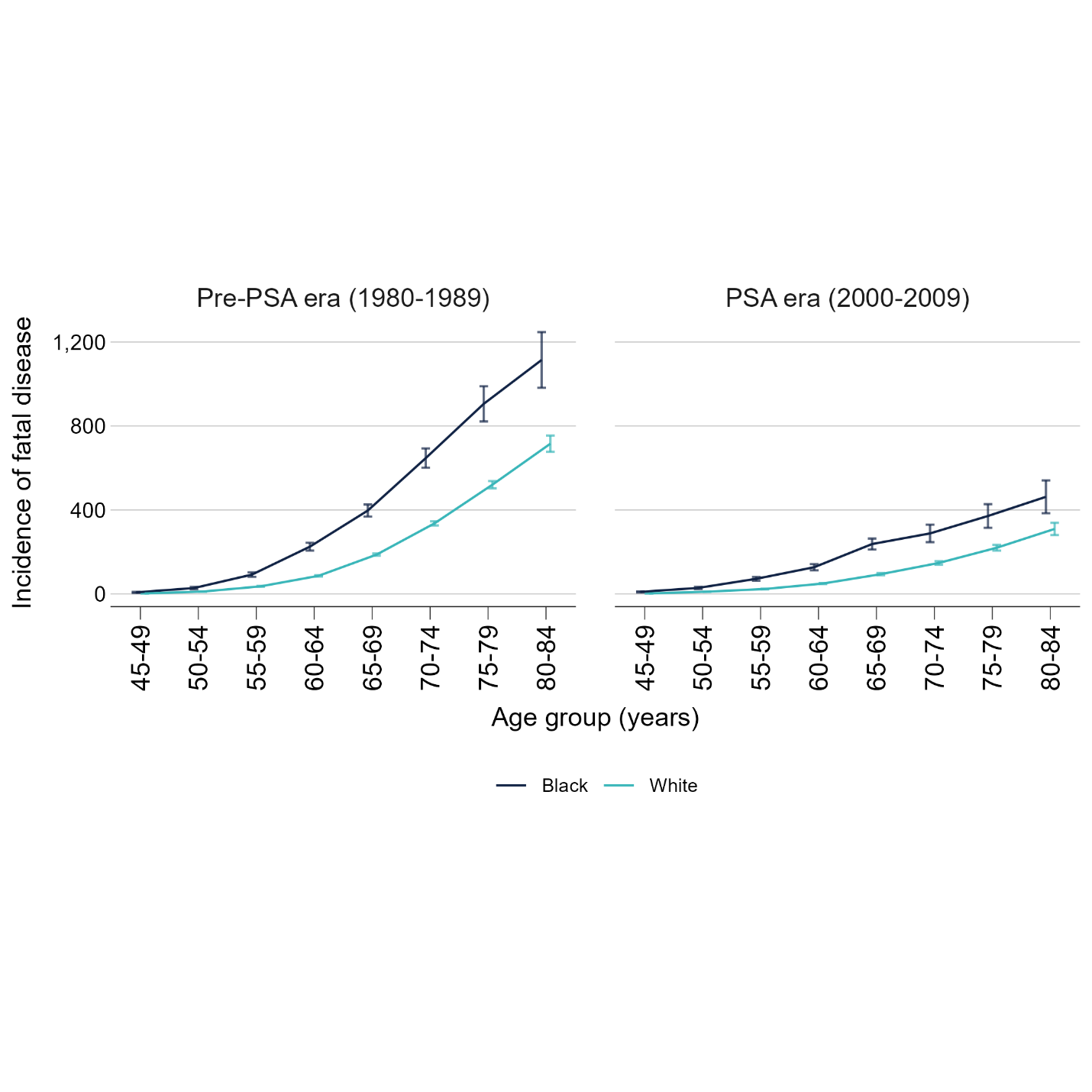


Annual incidence rates of fatal prostate cancer within 17 years of diagnosis by era, race, and age group. Figure shows observed incidence rates with 95% confidence intervals.

## Figure S5


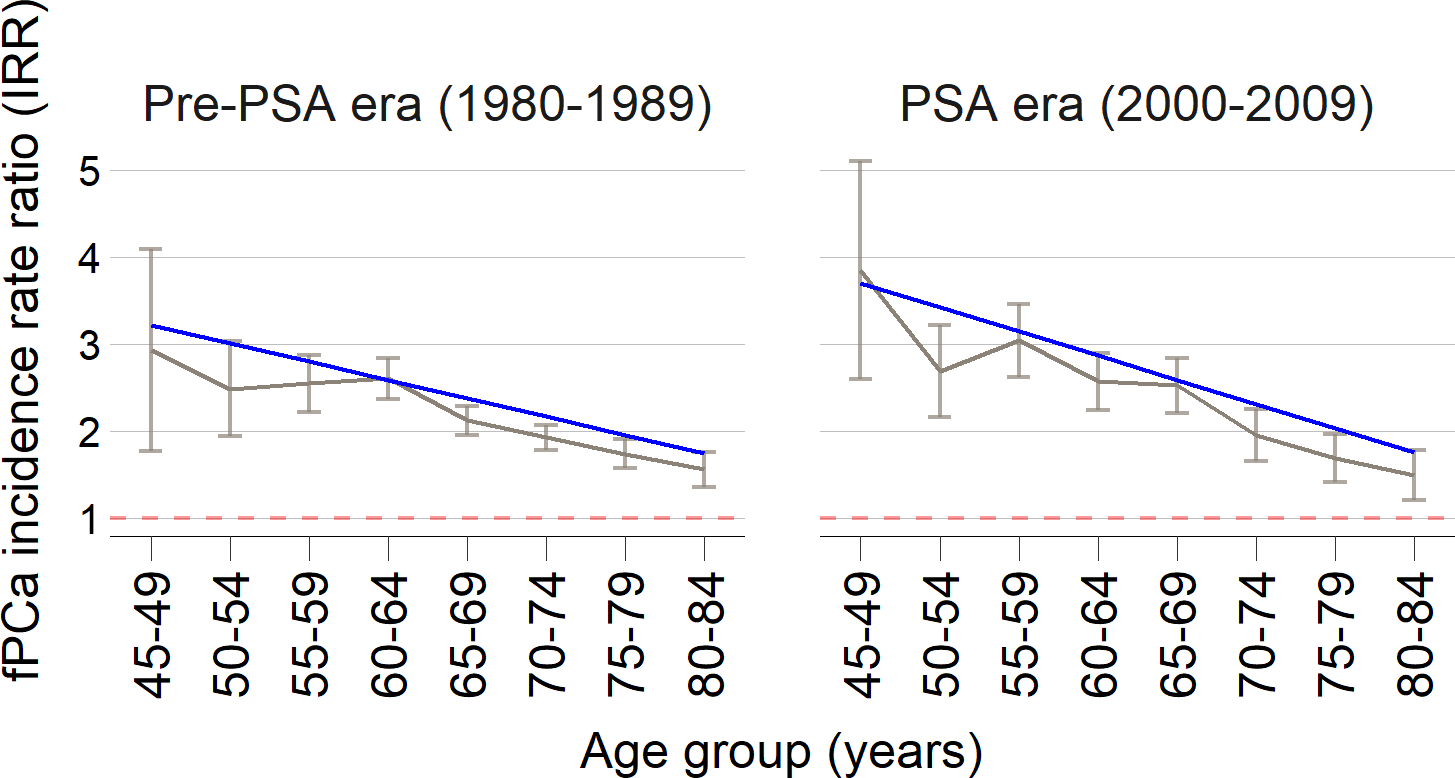


Black-to-White annual incidence rate ratios of fatal prostate cancer within 17 years of diagnosis by era and age group. Figure shows observed rate ratios with 95% confidence intervals and a fitted trend line based on weighted linear regression.

## Table S1

Fitted linear regression of Black-to-White incidence rate ratios of fatal prostate cancer within 17 years of diagnosis by age group at diagnosis and calendar period. Reference categories: 45-49 age group, and the pre-PSA era.

| **Parameter** | **Coefficient** | **95% CI** | **P-value** |
| --- | --- | --- | --- |
| Intercept | 3.221 | 2.888 to 3.554 | <0.001 |
| Age group | -0.211 | -0.266 to -0.155 | <0.001 |
| Era | 0.482 | -0.065 to 1.029 | 0.079 |
| Age group × era | -0.067 | -0.160 to 0.025 | 0.138 |

1. Cox C. Delta method. In: *Encyclopedia of Biostatistics*. Vol 6.; 2005. doi:[10.1002/0470011815.b2a15029](https://doi.org/10.1002/0470011815.b2a15029) [↑](#footnote-ref-1)
